# Population or family history based BRCA gene tests of breast cancer? A systematic review of economic evaluations

**DOI:** 10.1186/s13053-021-00191-0

**Published:** 2021-08-28

**Authors:** Zahra Meshkani, Ali Aboutorabi, Najmeh Moradi, Mostafa Langarizadeh, Ali Ghanbari Motlagh

**Affiliations:** 1 Department of Health Economics, School of Health Management and Information Sciences, Iran University of Medical Sciences, Tehran, Iran; 2 Health Management and Economics Research Center, School of Health Management and Information Sciences, Iran University of Medical Sciences, Tehran, Iran; 3 Department of Health Information Management, School of Health Management and Information Sciences, Iran University of Medical Sciences, Tehran, Iran; 4 Clinical Oncology, Shahid Beheshti Medical University, Tehran, Iran

**Keywords:** Economic evaluation, Genetic test, BRCA, Breast cancer

## Abstract

**Background:**

Nearly 56% of at-risk carriers are not identified and missed as a result of the current family-history (FH) screening for genetic testing. The present study aims to review the economic evaluation studies on BRCA genetic testing strategies for screening and early detection of breast cancer.

**Methods:**

This systematic literature review is conducted within the Cochrane Library, PubMed, Scopus, Web of Science, ProQuest, and EMBASE databases. In this paper, the relevant published economic evaluation studies are identified by following the standard Cochrane Collaboration methods and adherence to the Preferred Reporting Items for Systematic Reviews and Meta-Analyses (PRISMA) statement reporting some recommendations for articles up to March 2020. Thereafter, the inclusion and exclusion criteria are applied to screen the articles. Disagreements are resolved through a consensus meeting. The Consolidated Health Economic Evaluation Reporting Standards (CHEERS) checklist is used in the evaluation of quality. Finally, a narrative synthesis is performed. To compare the different levels of incremental cost-effectiveness ratio (ICER), the net present value is calculated based on a discount rate of 3% in 2019.

**Results:**

Among 788 initially retrieved citations, 12 studies were included. More than 60% of the studies were originated from high-income countries and were published after 2016. It is noteworthy that most of the studies evaluated the payer perspective. Moreover, the robustness of the results were analyzed through one-way and probabilistic sensitivity analyses in nearly 66% of these studies. Nearly, 25% of the studies are focused and defined population-based and family history BRCA tests as comparators; afterwards, the cost-effectiveness of the former was confirmed. The highest and lowest absolute values for the ICERs were $65,661 and $9 per quality adjusted life years, respectively. All studies met over 70% of the CHEERs criteria checklist, which was considered as 93% of high quality on average as well.

**Conclusions:**

The genetic BRCA tests for the general population as well as unselected breast cancer patients were cost-effective in high and upper-middle income countries and those with prevalence of gene mutation while population-based genetic tests for low-middle income countries are depended on the price of the tests.

**Supplementary Information:**

The online version contains supplementary material available at 10.1186/s13053-021-00191-0.

## Background

Breast cancer was attributed to 11.7% (2,261,419 cases for both sexes and all ages) of all cancer types, in 2020 globally [1]. At the same time period, the number of 7.8 million women who were diagnosed with the disease were alive in the past 5 years. Breast cancer has been introduced as the most prevalent cancer with more lost disability-adjusted life years (DALYs) rather than the other type of cancer in the world since 2020 [2].

If one only concentrates on the costs of the disease, over 77% of its costs are related to lost production and indirect costs. Direct medical expenses are accounted for nearly 19% of the costs, with chemotherapy having the highest share of the costs [3, 4].

Several studies have focused on the treatment costs of breast cancer. Based on Allaire et al. study, the treatment excess^1^cost of the disease for stages I to III/IV was ranged from $72,177 to $131,812 for women between 21 and 44 years old; while it was ranged from $53,288 to $124,237 for women between 45 and 64 years old within 12 months (from 2003 to 2010) in the North Carolina cancer registry, respectively. Higher breast cancer treatment costs for young women were attributed to higher prevalence as well as later-stage disease [5]. The minimum, maximum, and average direct medical costs of the disease in Italy were estimated at €6692, €12,825, and €10,970 between 2007 and 2011, respectively which the higher one was related to metastatic patients [6]. A study was conducted in 13 provinces of China to estimate the medical as well as non-medical expenditure of breast cancer in 2012 and lasted till 2014. On average, medical and non-medical, and the total expenditures were $7527, $922, and $8450, in US dollars, respectively. The highest expenditure was related to stage IV [7]. All of these studies demonstrated that breast cancer treatment costs were increased in advanced stages constantly; however, it can be reduced by early detection.

Early detection is possible by breast self-assessment as well as clinical breast cancer screening such as mammography [8] although genetic testing is considered as a new advances in medicine which urge women having an FH of breast cancer [9]. It was estimated that nearly 20% of the diseases were attributed to heredity and gene mutation [10–12].

Although several genes are attributed to breast cancer, the BRCA genes have been recognized as the most important ones. The risk of developing breast cancer in women who carry the BRCA is considered as 65%, compared with 12% in the general population [13, 14]. The risk assessment estimating, managing the preventive interventions are the advantages of BRCA tests for women having an FH of breast cancer, and it avoids a second surgery or having a risk-reduction surgery performed parallel to the treatment surgery for women with the affected breasts [15].

Two screening strategies were identified for breast cancer genetic testing including population-based testing and the FH-based genetic tests, especially for the BRCA genes. For the first one, the genetic tests were offered to all women at age 30 and older while for the second one the genetic tests were undergone only for women that fulfill the clinical criteria (the genetic BRCA tests for whom > = 10% with the probability for gene mutation) [16]. It was shown that more than 50% of the BRCA carriers are identified and missed by using the FH-based genetic tests strategy since numerous unaffected BRCA carriers do not fulfill the current clinical criteria threshold which is attributed to paternal inheritance, smaller nuclear and poor communication among families, lack of awareness of the FH of breast or ovarian cancers as well as other types of cancers, inability to access health records, pure chance, and population migration [14, 17, 18]. In spite of the limitations of the FH-based genetic tests to detect more at-risk women, it is still the current model in hospitals or specialized genetic clinics. The BRCA mutations are available for the general population and more women at risk of breast cancer will be identified by performing population-based testing [19].

To improve population health as well as efficient allocation of limited health care resources across interventions, health economic assessment of interventions is extremely critical. By this means, economic evaluation is one of the most common tools which guide health policymakers to choose the best strategy through evaluating and comparing the effectiveness and costs of different health interventions. The economic evaluations outcome which is determined by dividing the difference in costs by the difference in effects between strategies is considered as the incremental cost-effectiveness ratio (ICER). This index is compared and if it is lower than the threshold, it means that the intervention is cost-effective [20].

Based on the results of economic evaluation studies, the population-based BRCA tests were cost-effective compared to the FH- based genetic tests [17, 18, 21, 22]. The cost-effectiveness of FH-based strategy in comparison with no testing is addressed in a number of studies; moreover, based on the results, it is considered as cost-effective as well [23–25]. A systematic review has been carried out regarding the economic evaluation of genetic tests programs for breast and ovarian cancers in 2016. Based on the results, the FH-based screening was very cost-effective potentially and the population-based BRCA tests screening provided reasonable value economically among Ashkenazi Jews alone. The study recommended further studies on economic evaluation on genetic tests [26].

Although both strategies are cost-effective, to decrease breast cancer cases, an appropriate strategy (mass or selective screening) is not clear yet and needs more evidence [27]. The purpose of this study is to review the economic evaluation studies on the screening strategies of genetic BRCA tests for the early detection of breast cancer. Evidently, the results of this review can provide adequate information to help policymakers adopt more effective strategies by considering the resource limitations and reduce the burden of breast cancers in their societies as well.

## Methods

### Search strategy

This systematic review of the literature was performed to identify the relevant published economic evaluation studies of genetic screening BRCA tests of breast cancer following the standard Cochrane Collaboration methods as well as the adherence to the PRISMA statement reporting some recommendations in this regard [28].

The search was conducted using several databases, including the Cochrane Library, PubMed, Scopus, and Web of Science, ProQuest, and EMBASE databases for those articles published up to March 2020. The strategy terms involved some keywords or medical subject headings (MeSH). The used keywords for the search are the following:

“Economic Evaluation”, “Cost-Effectiveness Analysis”, “Cost-Benefit Analysis”, “Cost-Utility Analysis”, “Breast Neoplasm”, “Genetic Screening Testing”, “BRCA2 Protein”, “BRCA1 Protein”.

Specific search strategies are presented in Additional file 1. The protocol for this review was registered on PROSPERO in July, 2020 (The registered number is: CRD42020190811).

### Inclusion and exclusion criteria

Studies were selected based on the inclusion criteria as follows: Full-text studies; written in English addressed the areas of economic evaluations such as cost-effectiveness analysis (CEA), cost-utility analysis (CUA), cost-benefit analysis (CBA), and cost-minimization analysis (CMA) related to genetic screening BRCA tests of breast cancer. PICO has also been used as the inclusion criteria for this study as follows:

#### Population

All women affected or unaffected by gene mutations for breast cancer based on family history (high-risk and low-risk women in breast cancer), women without breast cancer or those who suffered from breast cancer as well as women in the general population were included in this study.

#### Interventions

Genetic testing for screening and early detection of breast cancer was considered as an intervention. All studies that have at least analyzed the BRCA genes in order to identify women at high risk for breast cancer were included.

#### Comparator

All the studies that considered the FH-based testing, as well as genetic tests for those women who were not selected as the arms in the model, were included in the study. Therefore, the following three types of comparators were considered: A) The FH-based genetic tests compared with no testing, B) Population-based as well as unselected women for genetic tests compared with no testing, and C) The FH-based genetic tests compared with population-based genetic tests as well as unselected women.

#### Outcomes

ICER, ICUR, Quality Adjusted Life Years (QALY), and Life Years Gain (LYG) were considered as the outcomes for genetic tests of breast cancer.

Studies that separately analyzed costs and effects, descriptive economic studies, cost-of-illness, the burden of disease, and economic burden, as well as micro-costing and economic evaluation studies, were excluded; moreover, they did not report the ICER or ICUR indices for genetic tests. The published studies that assessed the economic evaluations of preventive interventions for breast cancer such as mammography and MRI were also excluded.

### Study selection & data extraction

Duplicates citations have been removed using Endnote version 8. The title and abstract of the remained articles were screened by two reviewers (Z.M, A.A) independently based on the inclusion and exclusion criteria. Two independent reviewers (Z.M and N.M) were also responsible for screening the full text of the eligible articles. The articles in which did not meet the inclusion criteria were resolved by a consensus meeting by the third reviewer (A.GH). The list of references regarding eligible articles was manually checked to ensure the entry of all the studies related to the subject of the study.

The Cochrane Handbook for systematic review [29] was used to design the data collection form and the first authors, year of publishing, costing perspectives, interventions, population, ICER or ICUR indices, types of models (Markov or decision tree), and types of sensitivity analysis were considered for data extraction. The first author conducted data extraction and was checked by the second author.

### Quality assessment

Two reviewers (Z.M and N.M) independently assessed the quality of the selected studies, and the final quality assessment was evaluated based on consensus. The quality assessment was completed for each one of the included studies by the first author (Z.M). According to a review performed by Watts et al. in 2019, the most appropriate checklist for quality assessment of economic evaluation studies is the CHEERS checklist [30]. Accordingly, it includes five questions with 24 criteria in terms of title and abstract, introduction, methods, results, discussion, and conclusion [31]. Those studies which obtained 63 to 94% of the scores of the checklist were categorized into the group of high-quality [30].

The CHEERS checklist was used for quality assessment of the included studies and all the 24 criteria were scored in this regard. A score of quality assessment was given based on the percentage of criteria met by each study, which was ranged from 0 to 100%. For the items that completely covered the criteria, a perfect score (= 1) was allocated; for items that had an incomplete coverage of criteria, half of the score (= 0.5) was considered, and for those that did not cover the criteria, a score of zero was assigned, which were identified by ✓, #, and ×, respectively. As a result, the sum and percentage of quality assessment of the articles were calculated based on 24 points criteria.

### Data analysis

Due to the inconsistency as well as the heterogeneity of the studies in terms of the participants and the comparators, the meta-analysis could not be performed. Based on the extracted information, the characteristics, the conclusions, the reported outcome measures of all studies, and analytical approaches of the studies were summarized. Preferably, a narrative synthesis was performed.

In many studies, the costs and effectiveness of the interventions are examined at different time periods. Considering the inflation factor, they ought to be discounted to be comparable. By using the discounted rate, one can calculate and compare the true value of the cost-effectiveness ratio at a certain time period [27]. To compare the different levels of ICERs for the selected studies, the net present value of ICERs was calculated based on a discount rate of 3% in 2019, since it was the most updated data of the selected studies. The ICERs were converted to US dollars as well. The following formula was used to adjust the ICERs:

$$ \mathrm{NPV}=\mathrm{ICER}/\left(\left(1+\mathrm{r}\right)\hat{\mkern6mu} \left(2019-\mathrm{t}\right)\right) $$

Where NPV represents the net present value, r stands for the discount rate (=3% due to a high frequency in the selected studies), and t is designated as the studies’ data gathering time.

## Results

The literature search was conducted, and 788 articles were collected, 84 studies were obtained from PubMed, 75 from Embase, 403 from Scopus, 166 from ProQuest, 59 from WOS, and 1 from Cochrane. After removing 200 duplicates, 588 titles and abstracts were screened for eligibility, of which 39 articles were considered eligible after the full-text eligibility screening process. Accordingly, 21 articles were excluded due to assessing the cost-effectiveness of ovarian cancer as well as screening methods of breast cancer; moreover, two articles due to focusing on multigene tests, and four articles due to not reporting the ICERs indices, as well as low quality, were disregarded. Finally, 12 articles were included in this study.

**Fig. 1:**
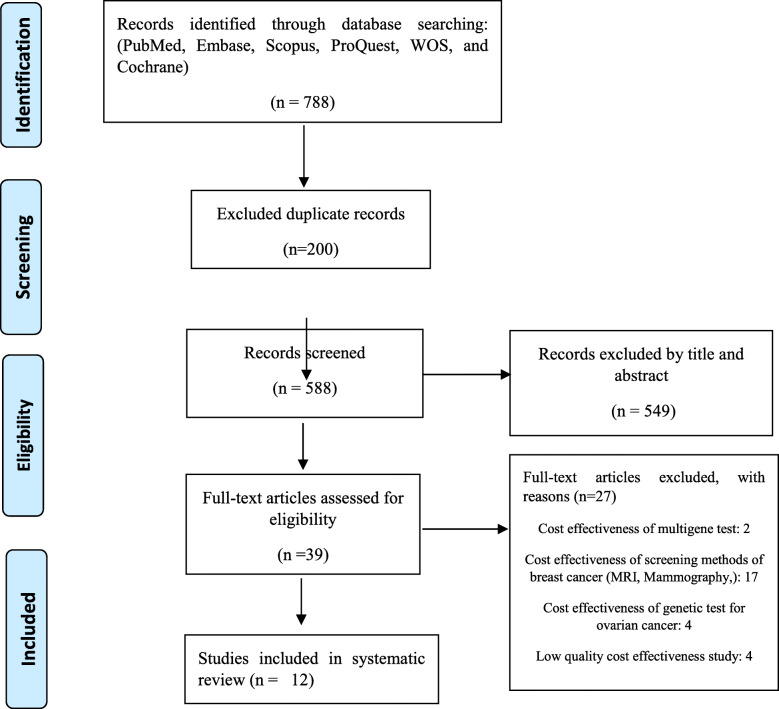
the PRISMA flow diagram for the study selection

Two reviewers (Z.M, A.A) applied the inclusion and exclusion criteria independently to screen the titles and abstracts of the identified articles. Disagreement during the selection of studies was 4.2% (of 788 articles) and the Kapa coefficient was 0.528. There has also been a disagreement only in the first step of study selection; moreover, for the remained stages, the authors agreed to the terms of the articles included in the study. Study selection based on the PRISMA diagram is shown in Fig. 1.

### Characteristics of studies

The economic evaluation studies of the genetic BRCA tests were mainly originated from the UK and US (58% of the studies), Spain, Norway, Australia, and Germany. In a study, India, Brazil, and the Netherlands were considered as low-middle and upper-middle-income countries [17].

Nearly 83% of the studies were published after 2016 and the oldest one was published in 2009. The selected studies had some differences in participants. Affected and unaffected women, those having an FH of breast and ovarian cancer, women with a family risk of cancer, breast cancer patients, and women from the general population were considered as the participants of this study.

Only four (33% of all) studies defined population-based and FH-based BRCA tests as comparators, while the others (= 8) used population-based or FH BRCA tests with no testing as well as the current criteria as comparators.

Among the selected studies, two studies assessed panel genetic tests, BRCA1/BRCA2/PALB2, and BRCA1/BRCA2/RAD51C/RAD51D/BRIP1/PALB2 as the intervention [9, 32], while the remaining ones (*n* = 10) assessed BRCA1/2 tests, only.

In the economic evaluation studies, at first, one needs to specify the point of view of the study (health system, payer, or social) in order to identify the exact costs and effectiveness of an intervention. Generally, the aforementioned perspectives are used to examine the costs and benefits. Although the researchers analyzed the costs in all types of the perspective (the health system, payer, or social) in the selected studies, most of them (58% of the studies) evaluated the payer perspective for their studies. The perspective of four studies (33% of the studies) was the health system and only one study did not state the cost perspective [21].

Both the currency and year for unit values were acknowledged only in five selected studies (41% of the studies) and the remaining ones only pointed out one of them [11, 17, 26–28]. The currencies that were mostly used were the US Dollar and Euro (*n* = 7) followed by the Brazilian currency (*n* = 1), Malaysian Ringgit (n = 1), Norwegian krone (n = 1), Australian Dollar (n = 1), and German Euro (n = 1).

All the studies were discounted costs and effectiveness was considered in the same count. Correspondingly, costs and effectiveness were discounted by 3 and 3.5% in five and six studies, respectively. In addition, the highest discount rate was 5% only in one study [23].

In the economic evaluation studies, the sensitivity analysis is conducted via examining the results by changing the parameters. It is possible to change all the parameters or some of them simultaneously; consequently, the results are examined and compared with the primary outcomes.

Sensitivity analysis was used for testing the robustness in all the included studies, along with both one-way and probabilistic sensitivity analyses (PSA) which were used in 66% of the studies, the PSA analysis alone in 25% of the studies, and univariate analysis was used only in one study [33], respectively.

All the studies used the cost-effectiveness analysis. Moreover, the difference in effects and costs for calculating the ICER index was not stated in the four selected studies [21, 32–34], while the remaining ones (66% of all) showed the difference in effects and costs for the index.

The outcomes of nearly 91% of studies were reported in the form of QALYs and only in one study, the outcomes were presented in LYGs form; however, two studies were used both the QALY and LYG as outcomes. The characteristics of these studies are summarized in Table 1.

**Table 1:** General characteristics of economic evaluation studies in genetic testing programs

Row	First author, Year	Country	Clinical condition	Participants (number)	Perspective	Intervention and Comparator (control)	Kind of EV	Type of model	Discount rate (%)	Time horizon	Sensitivity analysis	ICER/ ICUR	Conclusion
1	Manchanda, 2020 [17]	UK/USA/Netherlands/China/Brazil/ India	BC/OC	Women > = 30 years old	Payer and social	Population-based BRCA testing compared with FH based testing.	CEA	Markov model	3	Lifetime	One way-PSA	From a societal perspective ICER for UK, USA, Netherlands, China, Brazil, and India were $-5639, $-4018, $-11,433, $18,066, $13,579, and $23,031 per QALY, respectively. From a payer perspective ICER for UK, USA, Netherlands, China, Brazil, and India were $21,191, $16,552, $25,215, $23,485, $20,995, and $32,217 per QALY, respectively.	The population based BRCA testing for high as well as upper-middle income countries was cost-effective but it was not cost-effective in low-middle income countries in both payer and social perspective.
2	Correa-Galendi, 2020 [23]	Brazil	BC/OC	Healthy but high risk women > = 30 years old	Health system	FH based BRCA testing compared with no testing	CEA	Markov model	5	Lifetime (extending to the age of 70 years old)	PSA	ICER = US$21,724 per QALY and US$24,405 per LYG.	BRCA tests were recommended for Brazilian women but further research is needed.
3	Sun, 2019 [32]	UK, US	BC	11,836 unselected patients with BC	Payer and social	BRCA1/BRCA2/PALB2 testing for all cases with BC compared with current BRCA testing based on clinical criteria or FH alone.	CEA	Markov model	3.5	Lifetime (extending to the age of 80 years old)	One-way, PSA	Payer perspective: £10,464/QALY and $65,661/QALY for UK and US, respectively. Societal perspective £7216/QALY and $61,618/QALY for UK and US, respectively.	Multigene testing for all unselected patients with BC as well as subsequent predictive/cascade testing of relatives compared with FH testing was extremely cost-effective for UK and US.
4	Müller, 2019 [24]	German	BC/OC	A number of 4380 women(> = 35-year-old) with a BRCA 1/2 mutation	The German statutory health insurance	Genetic BRCA 1/2 testing for high risk women compared with no testing	CEA	Decision tree & Markov model	3	Lifetime (extending to the age of 65 years old)	PSA	€17,027 per QALY; €22,318 per LYG	The genetic BRCA testing to high-risk women that have a FH of cancer was cost-effective.
5	Manchanda, 2018 [9]	US, UK	BC/OC	All Jewish women > = 30	Payer	Two strategies were considered: 1- The standard clinical criteria/FH-based BRCA testing compared with panel testing for BRCA1/BRCA2/RAD51C/RAD51D/BRIP1/PALB2 2- population testing for BRCA compared with RAD51C/RAD51D/BRIP1/PALB2 mutations	CEA	Decision tree	3.5	Lifetime	One-way, PSA	For UK: £7629.65 for the first strategy, £21,599.96 for the second strategy For US: $49,282.19/QALY for the first strategy, $54,769.78/QALY for the second strategy	Population-based multigene testing (BRCA/RAD51C/RAD51D/BRIP1/PALB2) compared with current policy (FH based testing) was the most cost-effective strategy.
6	Norum, 2018 [33]	Norway	BC	535 women with BC	Health care and social perspective	The traditional FH approach compared with testing all patients with BC	CEA	Decision tree	3	Lifetime (extending to the age of 70 years old)	Univariate	Health care perspective: €40,503/LYG Societal perspective: €5669/LYG	BRCA testing for all patients with BC compared with FH strategy was cost-effective.
7	Tuffaha, 2018 [25]	Australia	BC	Affected women with BC > =30	Payer	BRCA testing for affected women compared with no testing	CEA	Decision tree & Markov model	5	Lifetime (until the age of 90)	One-way, PSA	$18,900 per QALY	BRCA testing in affected women was cost-effective compared with no testing
8	Lim, 2018 [35]	Malaysia	BC	A number of 1000 early stage patient, with BC (age = 40) in a hypothetical situation	Health system	BRCA testing for BC patients compared with no testing	CEA	Decision tree & Markov model	3	Lifetime	One-way, PSA	US$2725/QALY	BRCA mutation testing was cost-effective for BC patients and it is worthwhile to reimburse the test for high-risk cases to manage the treatment.
9	Patel, 2018 [22]	US, UK	BC/OC	Sephardi Jewish women (> = 30) for population based and those with 10% mutation risk for FH test	Payer	Population-based BRCA1 testing, compared with FH testing as the current practice of clinical criteria	CEA	Markov model	3.5	Lifetime (up to 83 and 82 years for UK and US populations, respectively)	One-way, PSA	£67.04/QALY and $308.42/QALY for UK and US people, respectively.	Population-based BRCA1 testing was highly cost-effective than clinical criteria FH testing
10	Manchanda, 2017 [16]	US, UK	BC/OC	Women (> = 30) with 1 to 4 Ashkenazi –Jewish grandparents	Payer	BRCA test for AJ founder mutations were compared with FH-based clinical criteria	CEA	Decision tree	3.5	Lifetime horizon (up to 83 years old)	PSA	For UK: £ -2960, £- 2327, £ -1254 and £ 863/ QALY for 4, 3, 2 and 1 AJ grandparents, respectively. For U.S: $-19,587, $-16,788, $-12,013 and $-2542 / QALY for 4, 3, 2 and 1 AJ grandparents, respectively.	Population BRCA testing was cost-effective for US and UK with varying levels of AJ ancestry.
11	Manchanda, 2015 [21]	UK	BC/OC	Ashkenazi –Jewish women (> = 30) for population based and those with 10% mutation risk for FH test	NA	Population-based BRCA testing compared with FH testing	CEA	Markov model	3.5	Lifetime	One-way, PSA	£ -2079/QALY	Population-based BRCA compared with FH testing was cost-effective.
12	Holland, 2009 [34]	USA	BC	High risk women (35-year-old) with an associated FH of cancer or concerned about having the gene mutation.	Societal perspective	FH testing compared with no testing	CEA	Semi-Markov model	3	Lifetime (extending to the age of 70 years old)	On- way, PSA	$ 9 /QALY	Although the FH test was cost-effective, the results were most sensitive to utility after BRCA mutation and the discount rate. So, the further research was needed for the reliability of the results.

*BC* Breast Cancer, *OV* Ovarian Cancer, *FH* Family History

Depending on the goal of economic evaluation studies, a model was designed based on a decision tree or Markov model. In the current review, for the selected studies, the Markov model was frequently used (*n* = 5), especially for the studies that considered hypothetical scenarios as well as cohort studies. The decision tree was used just in three studies, and the remaining ones (*n* = 3) used both of them. It is noteworthy that a Semi-Markov model was used in one study [34]. Although all the selected studies included a figure for the models, four of them (33%) have completed an explanation regarding the model as well as parameters [9, 16, 21, 22].

Risk-reducing mastectomy (RRM) and Risk reduction salpingo-oophorectomy (RRSO) is preventive interventions for the BRCA carriers, which all the studies considered preventive surgeries in the models and costs. Coronary heart disease (CHD) and hormone replacement therapy (HRT), as the compliances after RRSO, were pointed out in three studies [9, 17, 22]. Just in two studies (16% of all) for women with the negative gene mutation, as well as the ones who did not perform the genetic tests, the intensified surveillance and standard care were considered as the branches in the models [23, 24].

Three (25% of all) studies considered the genetic testing of family members in the model since one of the important goals,as well as advantages of genetic test, is to define high risks among relatives and family members of breast cancer pathogenic carriers [25, 32, 33].

### ICER analysis

Among the 12 selected articles, nearly 19 analyses of ICERs were performed regardless of different strategies. By considering these different strategies, overall, 36 ICERs were calculated for different countries as well as different time intervals.

The ICERs results were compared to a willingness to pay (WTP) threshold for nearly 58% (*n* = 7) of the selected studies. In addition, four studies used the NICE threshold for comparing the ICERs results [9, 16, 21, 22]. The thresholds varied across different countries. The WTP thresholds of £30,000/QALY, $100,000/QALY, and AU$50,000 per QALY were used for UK, US, and AU, respectively.

In a study by Manchanda et al. in 2020, 3*GDP per Capita and GDP per Capita were used for UK, USA, Netherlands, China, Brazil, and India as thresholds [17].

The ICERs for all the selected studies were below the standard threshold. Sun et al. [32] and Holland et al. [34] reported the highest ($65,661/QALY) and lowest ($9/QALY) absolute values for ICER respectively which were related to the USA.

For two studies, the value of ICERs for different strategies were negative, showing the cost-saving of population (unselected) genetic tests compared with the current one (FH testing) [16, 17].

**Fig. 2:**
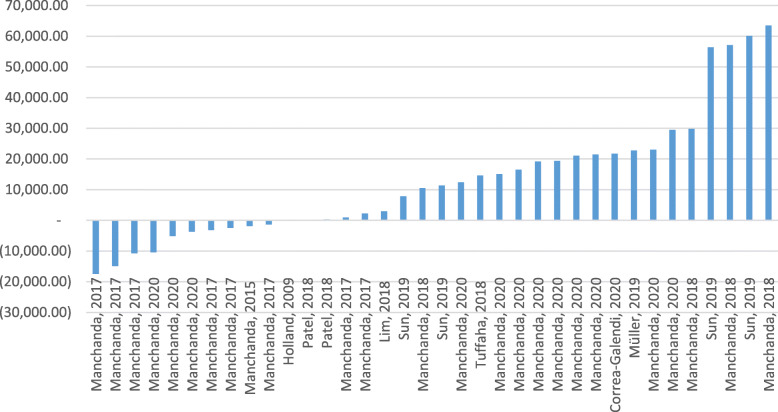
Adjusted ICERs for the selected studies

In the selected studies, population-based genetic tests were preferred compared with the FH-based testing [17, 22, 32], and FH-based testing was preferred compared with no testing [23–25, 34, 35]. Adjusted ICERs of the selected studies is shown in Fig. 2.

### Quality assessment

The quality assessment of the selected studies based on the CHEERS Checklist is presented in Table 2. All the selected studies met over 70% of the CHEERs criteria checklist. The average percent of the items reported in these studies was 93%. By considering the time cutoff point of 2018, higher scores were achieved for those recently published studies compared to the earlier ones (average scores of 95% vs 85%). Three studies met 100% of the CHEERs criteria. It is noteworthy that all the selected studies were of high quality. Perspective, time horizon, discount rate, measurement of effectiveness, model assumptions, and heterogeneity of explanation were the reasons for not achieving 100% of the criteria. In addition, three articles did not address the funding and one of them reported no conflict of interest.

**Table 2:** CHEERS checklist for quality assessment of the studies

Row	First author, Year	Title Identified as economic evaluation	Structured abstract	Intro provides context and a clear study question	Population characteristics	Setting and location	Study Perspective	Comparators described	Time horizon	Discount rate	Outcomes and relevance	Measurement of effectiveness	Pref based outcomes	Costs (unit costs and methods) or Cost model based studies	Currency, date and conversion	Model choice described	Model assumptions	Analysis methods	Parameters of values	Incremental costs	Sensitivity of incremental costs or model sensitivity analyses	Heterogeneity explained	Findings and limitations	Funding source	Potential conflict of interest	SORCE	Percent
1	Manchanda, 2020 [17]	✓	#		✓	✓	✓	✓	✓	✓	✓	✓	✓	✓	✓	✓	✓	✓	✓	✓	✓	✓	✓	✓	✓	23.5	97.91
2	Correa‑Galendi, 2020 [23]	×	✓	✓	✓	✓	✓	✓	✓	✓	✓	✓	✓	✓	✓	✓	✓	✓	✓	✓	✓	✓	✓	✓	✓	23	95
3	Sun, 2019 [32]	✓	✓	✓	✓	✓	✓	✓	✓	✓	✓	✓	✓	✓	✓	✓	✓	✓	✓	✓	✓	✓	✓	✓	✓	24	100
4	Müller, 2019 [33]	×	✓	✓	✓	✓	✓	✓	✓	✓	✓	✓	✓	✓	✓	✓	✓	✓	✓	✓	✓	✓	✓	✓	✓	23	95
5	Manchanda, 2018 [9]	✓	✓	✓	✓	✓	✓	✓	✓	✓	✓	✓	✓	✓	✓	✓	✓	✓	✓	✓	✓	✓	✓	✓	✓	24	100
6	Norum, 2018 [33]	✓	✓	✓	✓	✓	✓	✓	×	✓	✓	×	✓	✓	✓	✓	✓	✓	✓	✓	✓	✓	✓	✓	✓	22	92
7	Tuffaha, 2018 [25]	✓	✓	✓	✓	✓	✓	✓	✓	✓	✓	✓	✓	✓	✓	✓	×	✓	✓	✓	✓	×	#	×	✓	20.5	85
8	Lim, 2018 [35]	✓	✓	✓	✓	✓	✓	✓	✓	✓	✓	✓	✓	✓	✓	✓	✓	✓	✓	✓	✓	×	✓	✓	✓	23	95
9	Patel, 2018 [22]	✓	✓	✓	✓	✓	✓	✓	✓	✓	✓	✓	✓	✓	✓	✓	✓	✓	✓	✓	✓	✓	✓	✓	✓	24	100
10	Manchanda, 2017 [16]	✓	✓	✓	✓	✓	✓	✓	✓	✓	✓	✓	✓	✓	✓	✓	✓	✓	✓	✓	✓	×	✓	✓	✓	23	95
11	Manchanda, 2015 [21]	✓	✓	✓	✓	✓	×	✓	✓	✓	✓	✓	✓	✓	✓	#	×	✓	✓	✓	✓	✓	✓	✓	×	20.5	85
12	Holland, 2009 [34]	✓	✓	✓	✓	×	✓	✓	✓	✓	✓	✓	✓	✓	#	✓	✓	✓	✓	×	✓	×	#	×	×	18	75

✓ = 1, # = 0.5, × = 0

## Discussion

The current study reviewed 12 published economic evaluation studies conducted on the genetic tests for screening as well as early detection of breast cancer. The present study aimed to provide evidence for policymakers to have a cost-effective strategy for genetic tests, the FH or population-based genetic tests, to reduce the burden of breast cancer.

All the selected studies (100%) indicated that the genetic tests for early detection as well as risk management of breast cancer at the WTP thresholds were cost-effective and there were no challenges for confirming the cost-effectiveness of performing genetic tests.

Based on the results of our study, more than 96% of the selected studies, which assessed the population-based genetic tests and the FH-based tests as comparators, confirmed the cost-effectiveness of population-based BRCA tests for the general population as well as the unselected breast cancer patients [17, 22, 32]. Although the results of the study showed that population-based testing is more preferred compared to the FH- based testing, the second one was more cost-effective in comparison with no testing as well [18, 23–25, 34, 35].

The BRCA gene mutation in AJ and SJ is more frequent compared to other races. More than 50% of the selected studies were related to AJ and SJ women from the UK and US. The participants in Patel et al.’s study were all SJ women who make up nearly 20% of the UK population and based on the result, performing the genetic tests was considered as cost-effective [22]. In addition, Manchanda et al. have assessed the cost-effectiveness studies for AJ women, and this study was updated for the AJ population who married by non-Jews. These studies aimed to assess the cost-effectiveness of genetic tests by considering the different probabilities of BRCA mutations. They assessed the genetic tests for the population and also for women with 3, 2, or one Ashkenazi-Jewish grandparent in UK and U.S. Accordingly, these strategies were separately performed. Based on the results of population testing compared with the FH-based clinical criteria, they were highly effective for women who had two and one AJ grandparents in UK and US, respectively [16, 21].

Although the results of these studies confirmed the cost-effectiveness of the population-based testing compared with the FH-based testing, we cannot generalize these results for other races. Manchanda et al. assessed the genetic test strategies for high, upper-middle, and low-middle income countries. Based on the results, population-based testing was cost-effective for high and upper-middle-income countries. For high-income countries, it was cost-saving as well; while for India as a low-income country, the results were not similar, and the tests were not cost-effective in both payer and social perspectives unless the cost of the tests has changed to less than $172. The ICERs for high-income countries were negative and based on the health economics’ concept, the population-based genetic tests are considered as a dominant option due to a lower cost and higher effectiveness as well [17]. All the aforementioned findings show that regardless of the type of health system, as governmental or non-governmental, the population-based genetic test was cost-effective for high-income countries as well as those countries with a high prevalence of gene mutation.

Screening the family member for the breast cancer is the most advantageous of genetic tests, which was pointed out just in three studies [25, 32, 33] focusing on cascade testing. Sun et al. performed cascade testing for relatives of mutation carriers and based on the results, the multigene testing was found to be extremely cost-effective for all the unselected patients having breast cancer as well as subsequent predictive/cascade testing of relatives [32].

For women having positive BRCA genetic tests, risk reduction interventions (RRM and RRSO) were recommended; however, most of them, especially for women aged 35 years old, the interventions were postponed because of childbearing. Hence, they selected severe follow-up. In addition, sensitivity analysis was considered in some studies, and based on the results, the ICERs were not sensitive to postponing the surgical preventions [22, 25, 32, 34]. Besides the above-mentioned reasons, delaying surgery can also have cultural causes, which make people ashamed; thus they choose to take risks in this regard. Furthermore, lack of trust in physicians due to conflicting interests in performing surgery, lack of sufficient knowledge on the benefits of preventive measures, as well as treatment management can be considered as reasons for delaying or even not performing the needed surgeries. The price of the genetic tests and risk reduction interventions can also be considered as the most important factors for postponing the interventions which insurance coverage can help in reducing the costs for women who are willing to perform the interventions as Holland et al. and Lim et al. recommended the insurance coverage of genetic tests as well [34, 35]. Given that 5 to 10% of breast cancers occur because of gene mutations, insurance companies can make profits by risk pooling as well as preventing the disease severity in the long run. Insurance companies can adjust the premium as well as coverage based on age, race, and gene mutation prevalence variables.

All the sensitivity analyses confirmed the robustness of the results of the studies by performing one-way or the PSA. The prevalence of gene mutation [24] and the price of the test [33] were the parameters that affected the variability of the results. Discount rate, the probability of gene mutation, and types of prevention interventions were the other parameters assigned in sensitivity analysis; however, they did not change the result as the same rate as the gene mutations and the price of the tests [25].

One important step in economic evaluation studies is to define the perspective of the study, which is considered as payer, patient, or health system. If it is not identified or missed, the costs could not be addressed, and also outcomes may be over or underestimated. The majority of the selected studies defined the perspective of their study as payer; hence the costs consisted of direct medical costs as well as treatment costs in the model [9, 16, 17, 22, 24, 25, 32]. Norum et al. mentioned a comprehensive point to the costs and considered direct and indirect costs such as preventive interventions, transport, accommodation place, and lost production. Although the cost of loss of production because of performing the genetic tests can be ignored, it is important for preventive interventions [33]. However, the genetic screening test based on all types of perspectives was known as a cost-effective method.

Based on Holland et al. the results of ICER were most sensitive to the utility after the BRCA mutation and the discount rate. The study was performed in 2009, in which the technology was not developed at that time period [34]. Technology can make testing available to people on a large scale because it reduces the cost of service. Moreover, it can increase the demand even for low-income patients. Therefore, advances in technology can improve the supply and demand of testing, and as a result, mutual benefits for health as well as patients are provided.

The population and FH-based genetic tests were assessed in two studies by panel testing instead of the BRCA1/2, which both confirmed the cost-effectiveness of the tests for population-based strategy. Manchanda et al. compared population-based on the BRCA1/BRCA2/RAD51C/RAD51D/BRIP1/PALB2 tests between the affected and unaffected women having the current clinical tests, and the FH-based BRCA1/2 tests, in which population-based screening tests were defined as highly cost-effectiveness strategies in the UK and US population. Additionally, based on Sun et al. study, BRCA1/BRCA2/PALB2 testing for all cases having breast cancer were compared with the current BRCA testing based on the clinical criteria or the FH alone which were cost-effective [9, 32]. The new technologies for genetic tests are in progress as fast as possible and their demand are increased by improving the people’s knowledge on risk management of the disease. However, this mostly depends on their preference for performing the genetic tests in which policymakers should pay attention to all the aforementioned factors.

### Limitation

Due to the lack of evidence for the genetic BRCA test for breast cancer as well as the use of the test for early detection of ovarian cancer, we were unable to exclude those studies which pointed out ovarian cancer as well as panel testing for breast cancer for the review.

## Conclusions

The genetic BRCA tests are considered as cost-effective methods for the general population as well as unselected breast cancer patients in high and upper-middle-income countries, in which the prevalence of gene mutation is high. More than 50% of the studies were performed in societies in which genetic mutations were prevalent and as a result, the population-based BRCA screening tests were cost-effective. The result may be different from those studies performed in other societies as a result of differences in the genetic structure. The population-based genetic tests for low-middle-income countries depend on the price of the tests. Overall, cascade genetic testing was considered as a cost-saving as well as a cost-effective method for both strategies. As the medical sciences are developed further, it is considered that multigene tests are more cost-effective compared with the BRCA 1/2 genes although cost-effectiveness studies for population-based multigene tests should be performed to choose the best strategy in the future.

### Recommendation

In the next decade, the genetic testing will likely include multigene panels and multiple diseases, especially when applied to more or less unselected subjects. Therefore, it is recommended that the systematic review as well as the original genetic science studies and their economic impact on policy development ought to be updated.

## Supplementary Information

**Additional file 1.**

## Data Availability

All data generated or analyzed during this study are included in this published article.

## Abbreviations

PICO: Population, Interventions, Comparator, Outcomes
NPV: Net Present Value
WTP: Willingness To Pay

## Footnotes

1 Subtracting off the mean medical costs of the comparison population
