# Supplementary material for: Population or family history based BRCA gene tests of breast cancer? A systematic review of economic evaluations
Source: Hered Cancer Clin Pract. 2021 Aug 28;19:35. doi: 10.1186/s13053-021-00191-0 (PMC8399845; doi:10.1186/s13053-021-00191-0)

Scopus: 403

## TITLE-ABS-KEY ( "Cost-Benefit Analysis"  OR  "Cost-Benefit Analyses"  OR  "Cost-Benefit Analysis"  OR  "Cost-Benefit Analyses"  OR  "Cost-Effectiveness"  OR  "Economic Evaluation"  OR  "Economic Evaluations" )  AND  TITLE-ABS-KEY ( "Breast Neoplasm"  OR  "Breast Neoplasm"  OR  "Breast Tumors"  OR  "Breast Tumor"  OR  "Breast Cancer"  OR  "Mammary Cancer"  OR  "Mammary Cancers"  OR  "Malignant Neoplasm of Breast"  OR  "Breast Malignant Neoplasm"  OR  "Breast Malignant Neoplasms"  OR  "Malignant Tumor of Breast"  OR  "Breast Malignant Tumor"  OR  "Breast Malignant Tumors"  OR  "Cancer of Breast"  OR  "Cancer of the Breast" )  AND  TITLE-ABS-KEY ( "Genetic Testing"  OR  "Genetic Predictive Testing"  OR  "Predictive Genetic Testing"  OR  "Genetic Predisposition Testing"  OR  "Genetic Screening"  OR  "Genetic Screenings"  OR  "BRCA2 Protein"  OR  "BRCA2 Protein"  OR  "BRCA1 Protein"  OR  "BRCA1 Protein" )


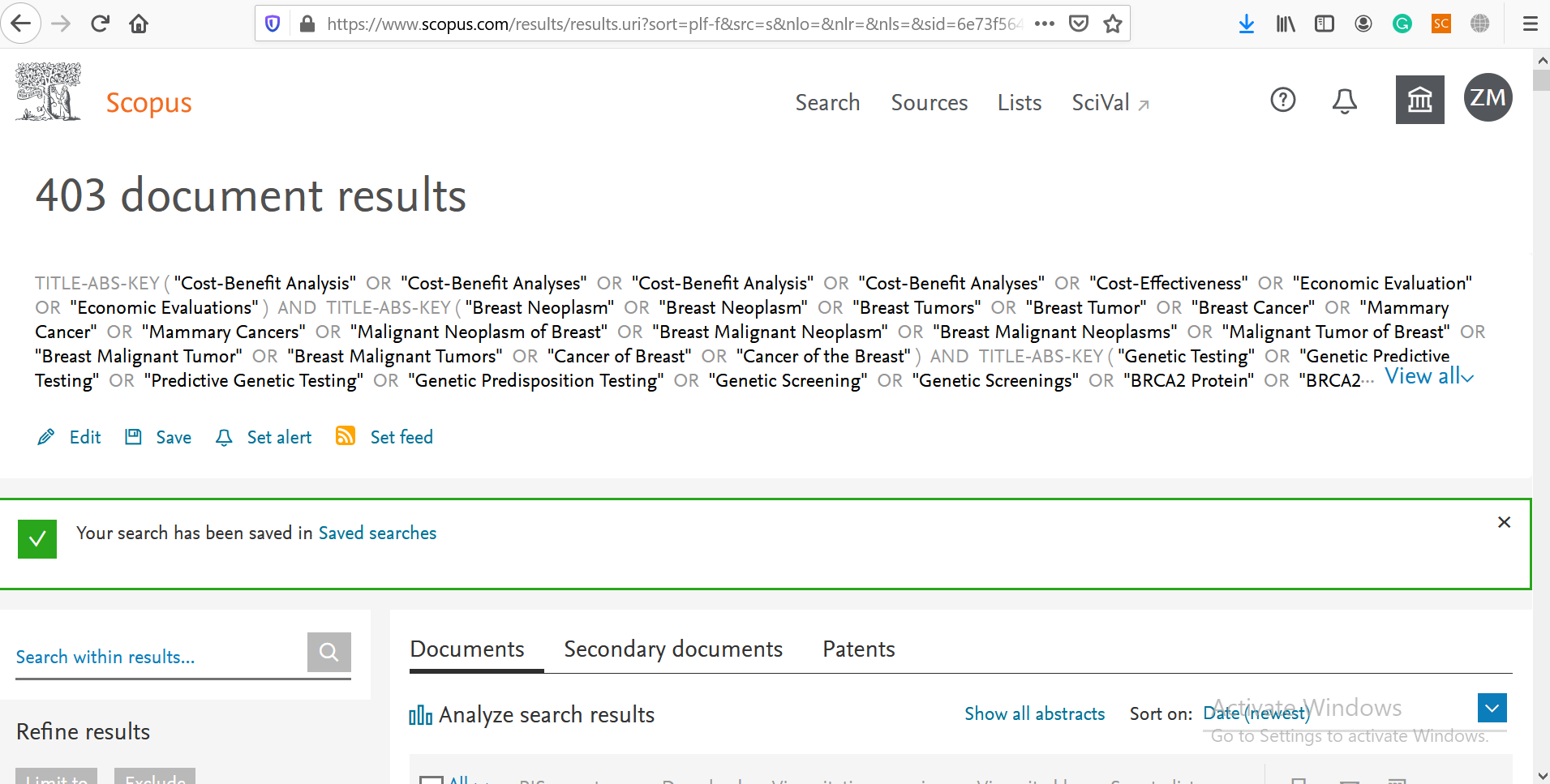


ProQuest =166

## TITLE-ABS-KEY ( "Cost-Benefit Analysis"  OR  "Cost-Benefit Analyses"  OR  "Cost-Benefit Analysis"  OR  "Cost-Benefit Analyses"  OR  "Cost-Effectiveness"  OR  "Economic Evaluation"  OR  "Economic Evaluations" )  AND  TITLE-ABS-KEY ( "Breast Neoplasm"  OR  "Breast Neoplasm"  OR  "Breast Tumors"  OR  "Breast Tumor"  OR  "Breast Cancer"  OR  "Mammary Cancer"  OR  "Mammary Cancers"  OR  "Malignant Neoplasm of Breast"  OR  "Breast Malignant Neoplasm"  OR  "Breast Malignant Neoplasms"  OR  "Malignant Tumor of Breast"  OR  "Breast Malignant Tumor"  OR  "Breast Malignant Tumors"  OR  "Cancer of Breast"  OR  "Cancer of the Breast" )  AND  TITLE-ABS-KEY ( "Genetic Testing"  OR  "Genetic Predictive Testing"  OR  "Predictive Genetic Testing"  OR  "Genetic Predisposition Testing"  OR  "Genetic Screening"  OR  "Genetic Screenings"  OR  "BRCA2 Protein"  OR  "BRCA2 Protein"  OR  "BRCA1 Protein"  OR  "BRCA1 Protein" )  AND  ( LIMIT-TO ( LANGUAGE ,  "English" ) )


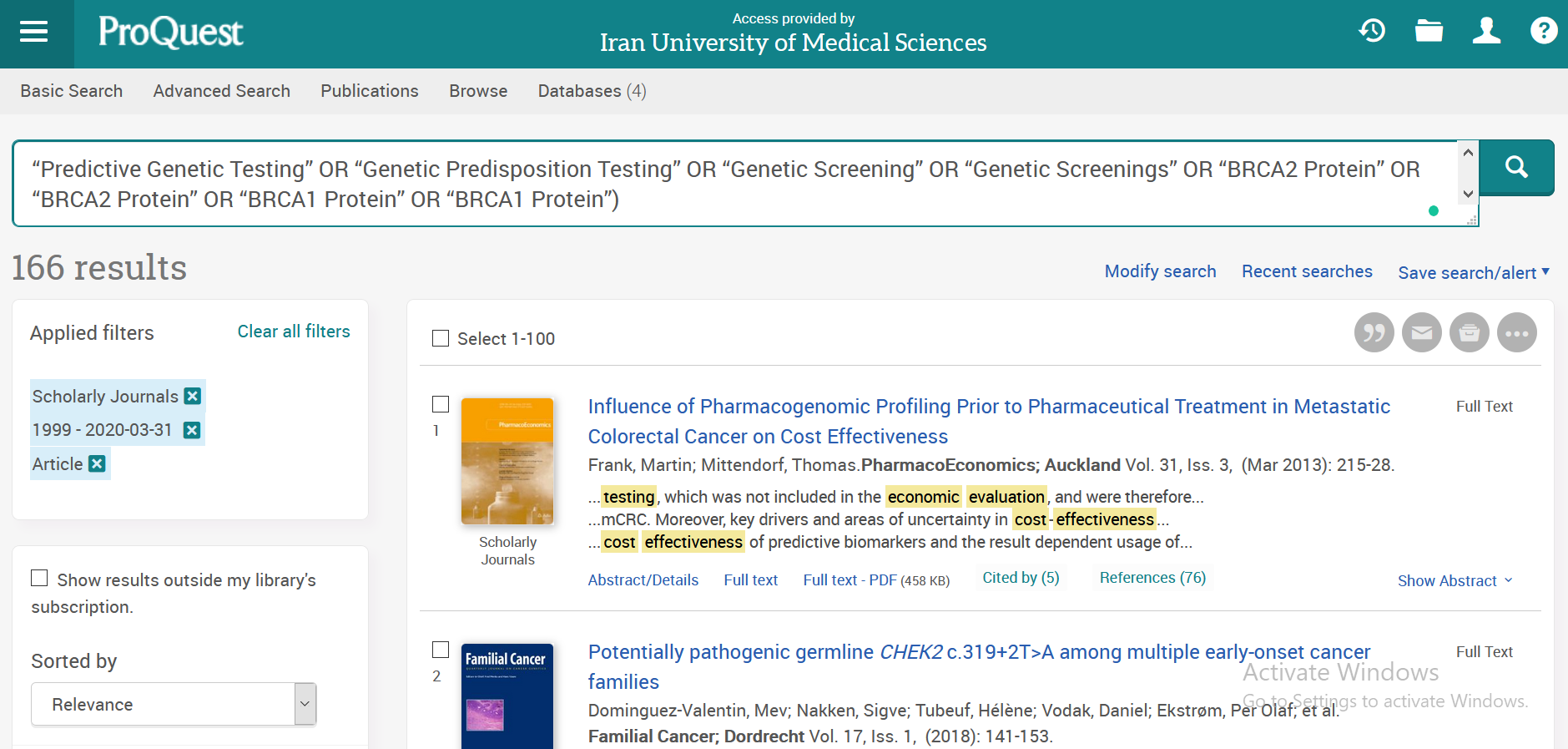


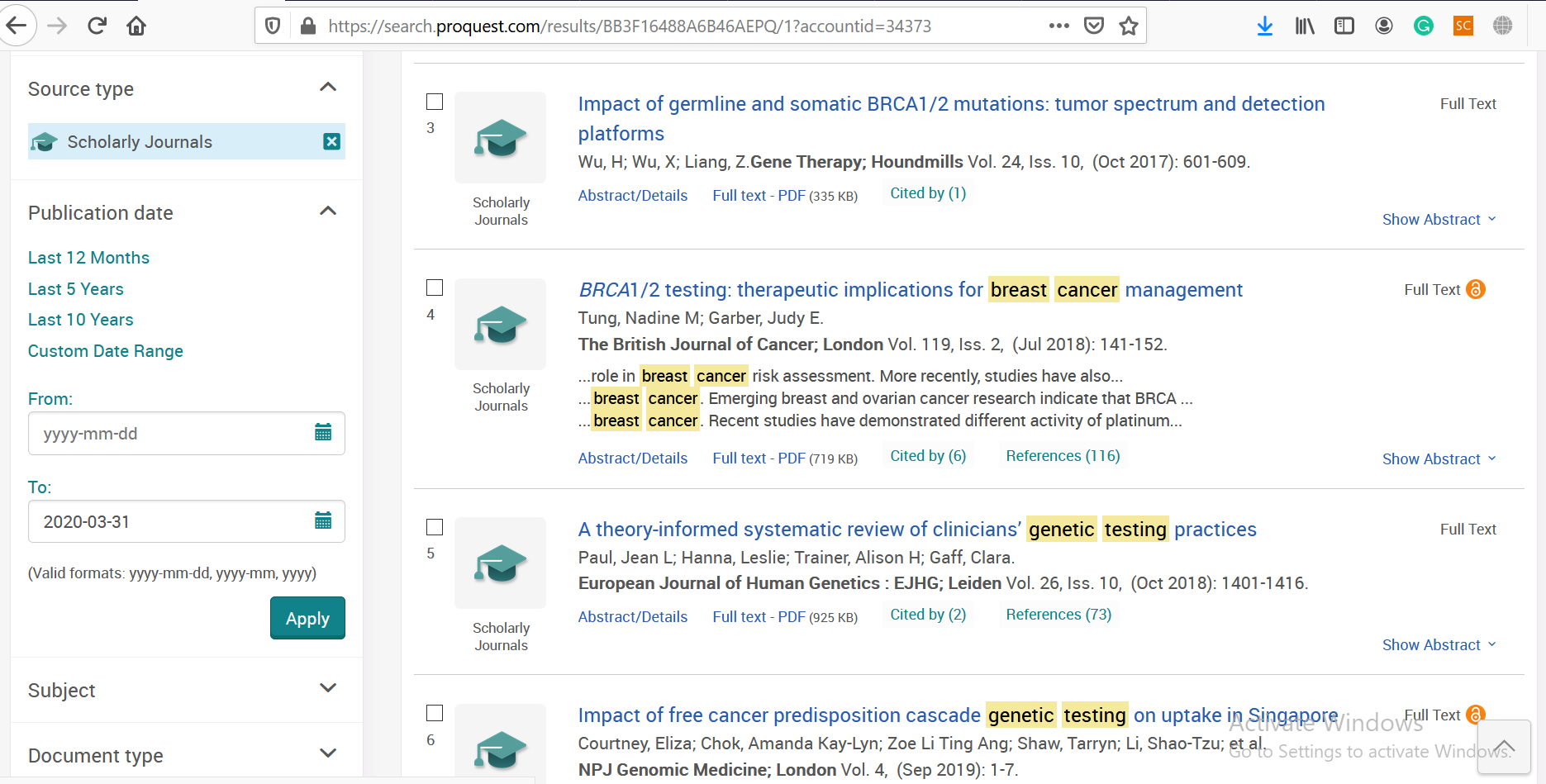


PubMed= 84

("Cost-Benefit Analysis"[MH] OR "Cost-Benefit Analyses"[TIAB] OR "Cost Benefit Analysis"[TIAB] OR "Cost Benefit Analyses"[TIAB] OR "Cost Effectiveness"[TIAB] OR "Economic Evaluation"[TIAB] OR "Economic Evaluations"[TIAB]) AND ("Breast Neoplasm"[MH] OR "Breast Neoplasm"[TIAB] OR "Breast Tumors"[TIAB] OR "Breast Tumor"[TIAB] OR "Breast Cancer"[TIAB] OR "Mammary Cancer"[TIAB] OR "Mammary Cancers"[TIAB] OR "Malignant Neoplasm of Breast"[TIAB] OR "Breast Malignant Neoplasm"[TIAB] OR "Breast Malignant Neoplasms"[TIAB] OR "Malignant Tumor of Breast"[TIAB] OR "Breast Malignant Tumor"[TIAB] OR "Breast Malignant Tumors"[TIAB] OR "Cancer of Breast"[TIAB] OR "Cancer of the Breast"[TIAB]) AND ("Genetic Testing"[MH] OR "Genetic Predictive Testing"[TIAB] OR "Predictive Genetic Testing"[TIAB] OR "Genetic Predisposition Testing"[TIAB] OR "Genetic Screening"[TIAB] OR "Genetic Screenings"[TIAB] OR "BRCA2 Protein"[MH] OR "BRCA2 Protein"[TIAB] OR "BRCA1 Protein" [MH] OR "BRCA1 Protein" [TIAB])


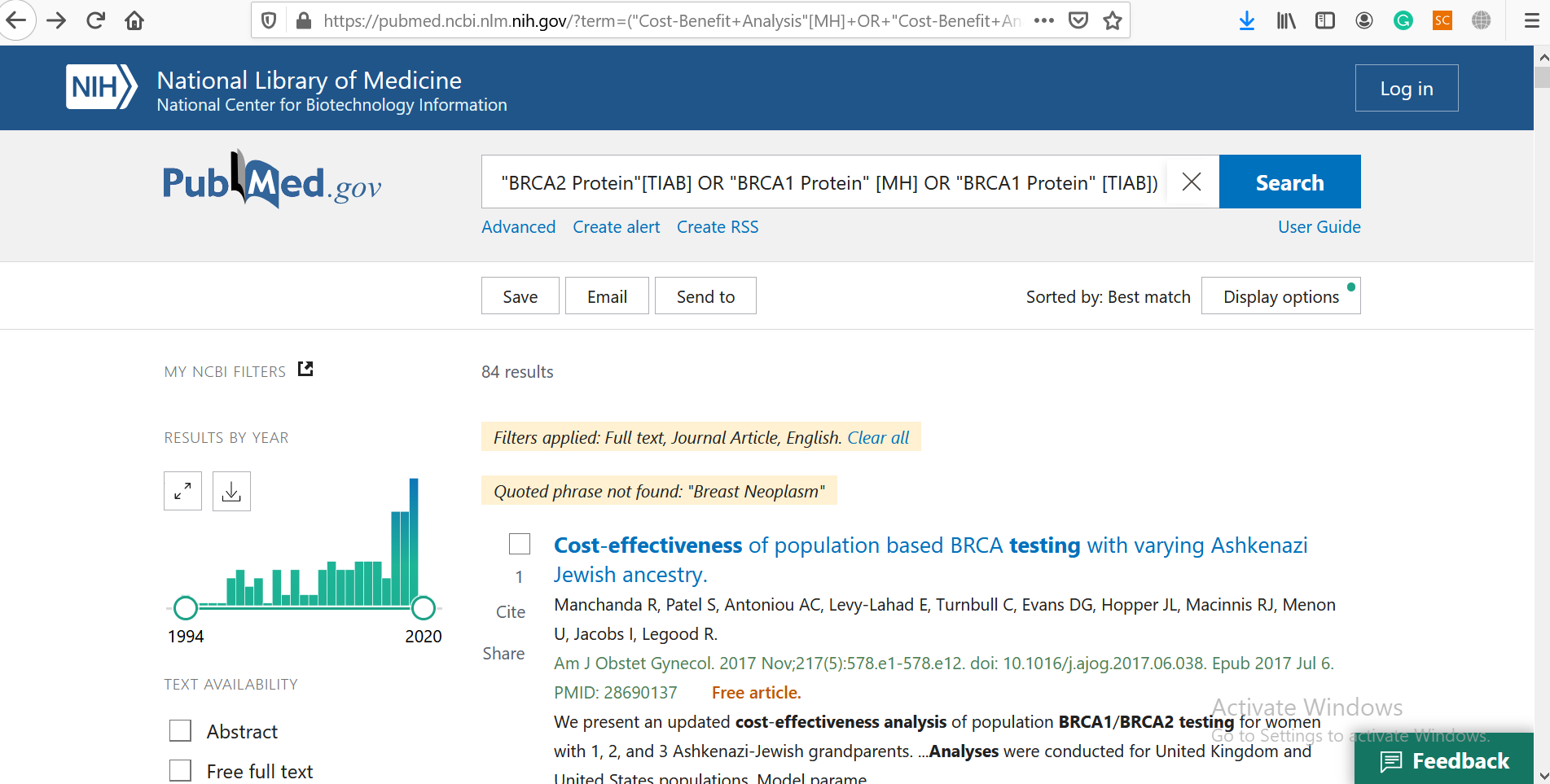


## WOS: 59

(TS=(“Cost-Benefit Analysis”  OR “Cost-Benefit Analyses”  OR “Cost-Benefit Analysis”  OR “Cost-Benefit Analyses”  OR “Cost-Effectiveness”  OR “Economic Evaluation”  OR “Economic Evaluations”)  AND TS=(“Breast Neoplasm”  OR “Breast Neoplasm”  OR “Breast Tumors”  OR “Breast Tumor”  OR “Breast Cancer”  OR “Mammary Cancer”  OR “Mammary Cancers”  OR “Malignant Neoplasm of Breast”  OR “Breast Malignant Neoplasm”  OR “Breast Malignant Neoplasms”  OR “Malignant Tumor of Breast”  OR “Breast Malignant Tumor”  OR “Breast Malignant Tumors”  OR “Cancer of Breast”  OR “Cancer of the Breast”)  AND TS=(“Genetic Testing”  OR “Genetic Predictive Testing”  OR “Predictive Genetic Testing”  OR “Genetic Predisposition Testing”  OR “Genetic Screening”  OR “Genetic Screenings”  OR “BRCA2 Protein”  OR “BRCA2 Protein”  OR “BRCA1 Protein”  OR “BRCA1 Protein”))  AND **LANGUAGE:** (English) AND **DOCUMENT TYPES:** (Article)

**Timespan:** All years. **Indexes:** SCI-EXPANDED, SSCI, A&HCI, CPCI-S, CPCI-SSH, BKCI-S, BKCI-SSH, ESCI, CCR-EXPANDED, IC.


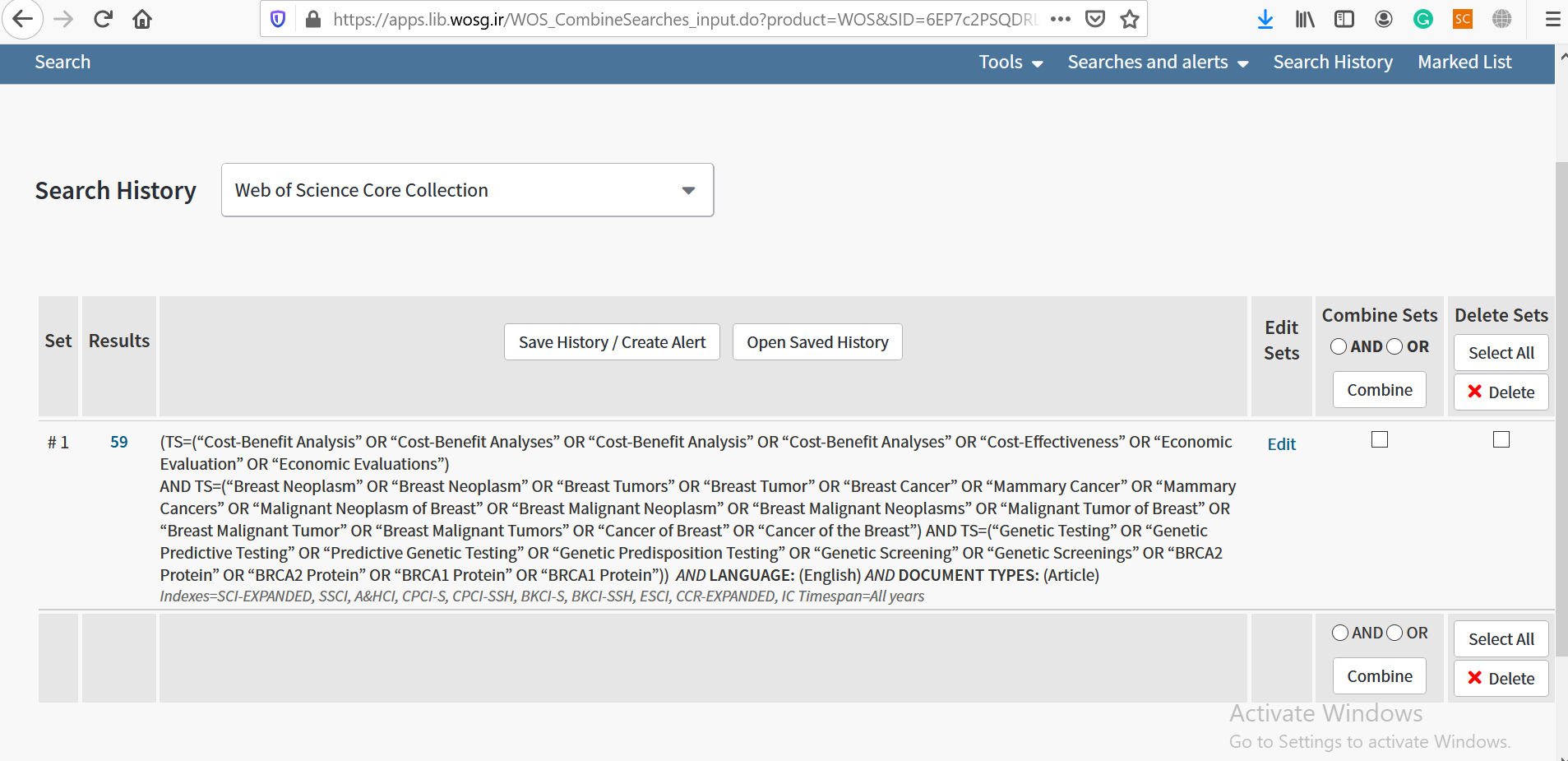


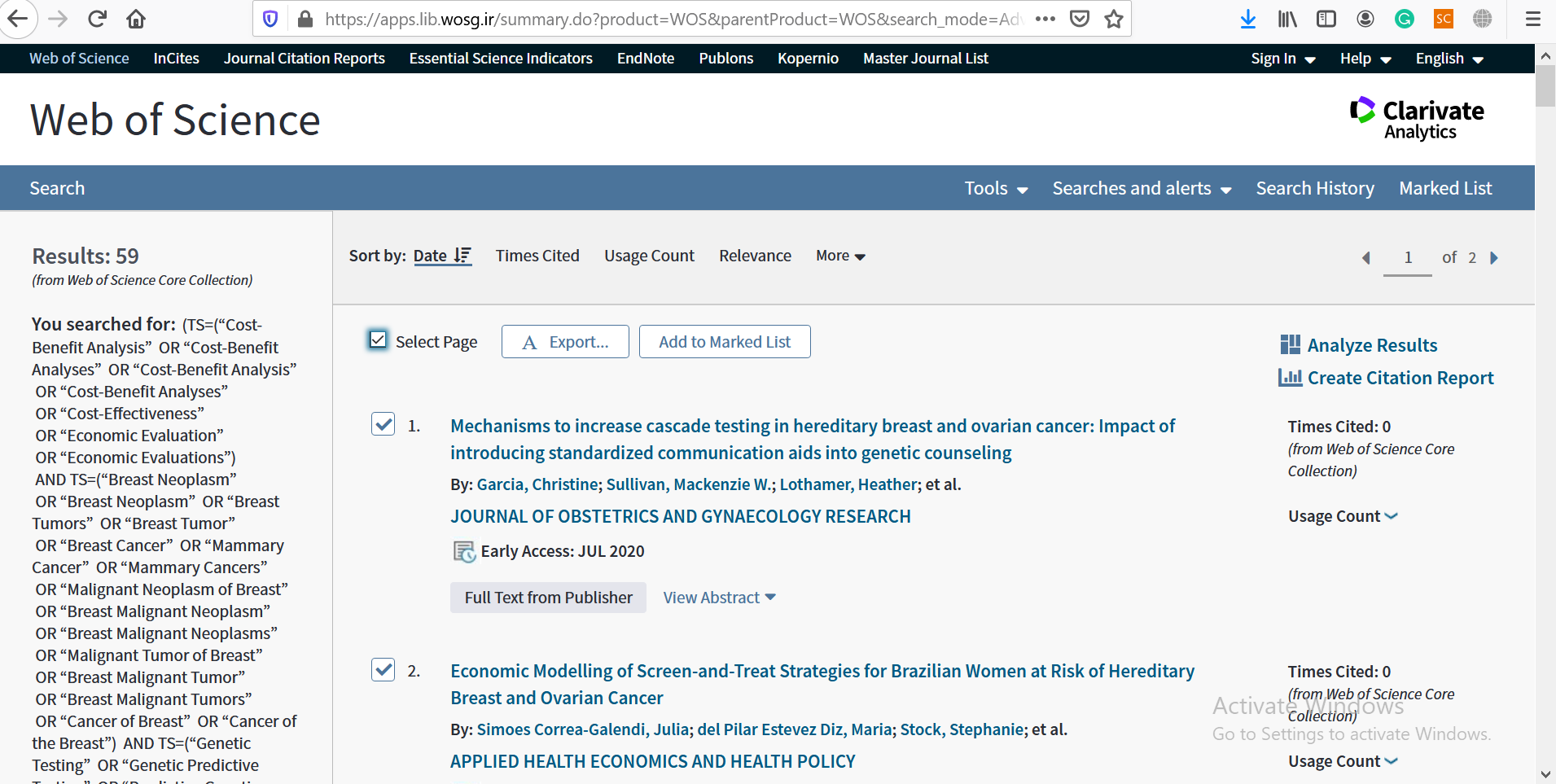


Embase: 75

(('cost-benefit analysis'/exp OR 'cost benefit analysis':ti,ab) AND 'cost-benefit analyses':ti,ab OR 'cost benefit analyses':ti,ab OR 'cost effectiveness':ti,ab OR 'economic evaluation':ti,ab OR 'economic evaluations':ti,ab) AND ('breast cancer'/exp OR 'breast cancer':ti,ab OR 'breast neoplasm':ti,ab OR 'breast tumors':ti,ab OR 'breast tumor':ti,ab OR 'mammary cancer':ti,ab OR 'mammary cancers':ti,ab OR 'malignant neoplasm of breast':ti,ab OR 'breast malignant neoplasm':ti,ab OR 'breast malignant neoplasms':ti,ab OR 'malignant tumor of breast':ti,ab OR 'breast malignant tumor':ti,ab OR 'breast malignant tumors' OR 'cancer of breast':ti,ab OR 'cancer of the breast':ti,ab) AND ('genetic screening'/exp AND 'genetic testing':ti,ab OR 'genetic predictive testing':ti,ab OR 'predictive genetic testing':ti,ab OR 'genetic predisposition testing':ti,ab OR 'genetic screenings':ti,ab OR 'brca2 protein':ti,ab OR 'brca1 protein':ti,ab)


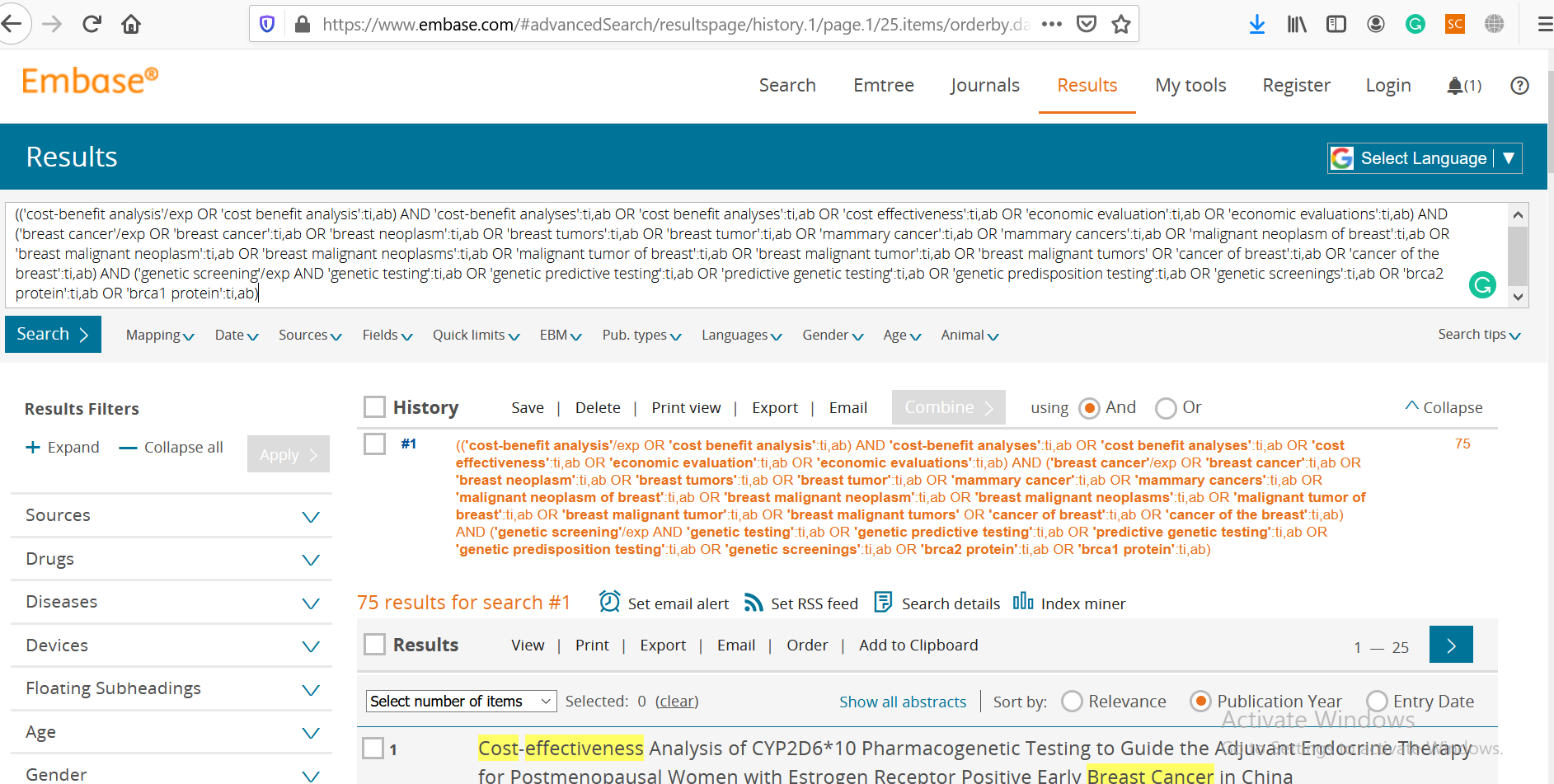


Cochrane: 1

## Title Abstract Keyword (“Cost-Benefit Analysis” OR “Cost-Benefit Analyses” OR “Cost Benefit Analysis” OR “Cost Benefit Analyses” OR “Cost Effectiveness” OR “Economic Evaluation” OR “Economic Evaluations”) AND Title Abstract Keyword (“Breast Neoplasm” OR “Breast Neoplasm” OR “Breast Tumors” OR “Breast Tumor” OR “Breast Cancer” OR “Mammary Cancer” OR “Mammary Cancers” OR “Malignant Neoplasm of Breast” OR “Breast Malignant Neoplasm” OR “Breast Malignant Neoplasms” OR “Malignant Tumor of Breast” OR “Breast Malignant Tumor” OR “Breast Malignant Tumors” OR “Cancer of Breast” OR “Cancer of the Breast”) AND Title Abstract Keyword (“Genetic Testing” OR “Genetic Predictive Testing” OR “Predictive Genetic Testing” OR “Genetic Predisposition Testing” OR “Genetic Screening” OR “Genetic Screenings” OR “BRCA2 Protein” OR “BRCA2 Protein” OR “BRCA1 Protein” OR “BRCA1 Protein”)


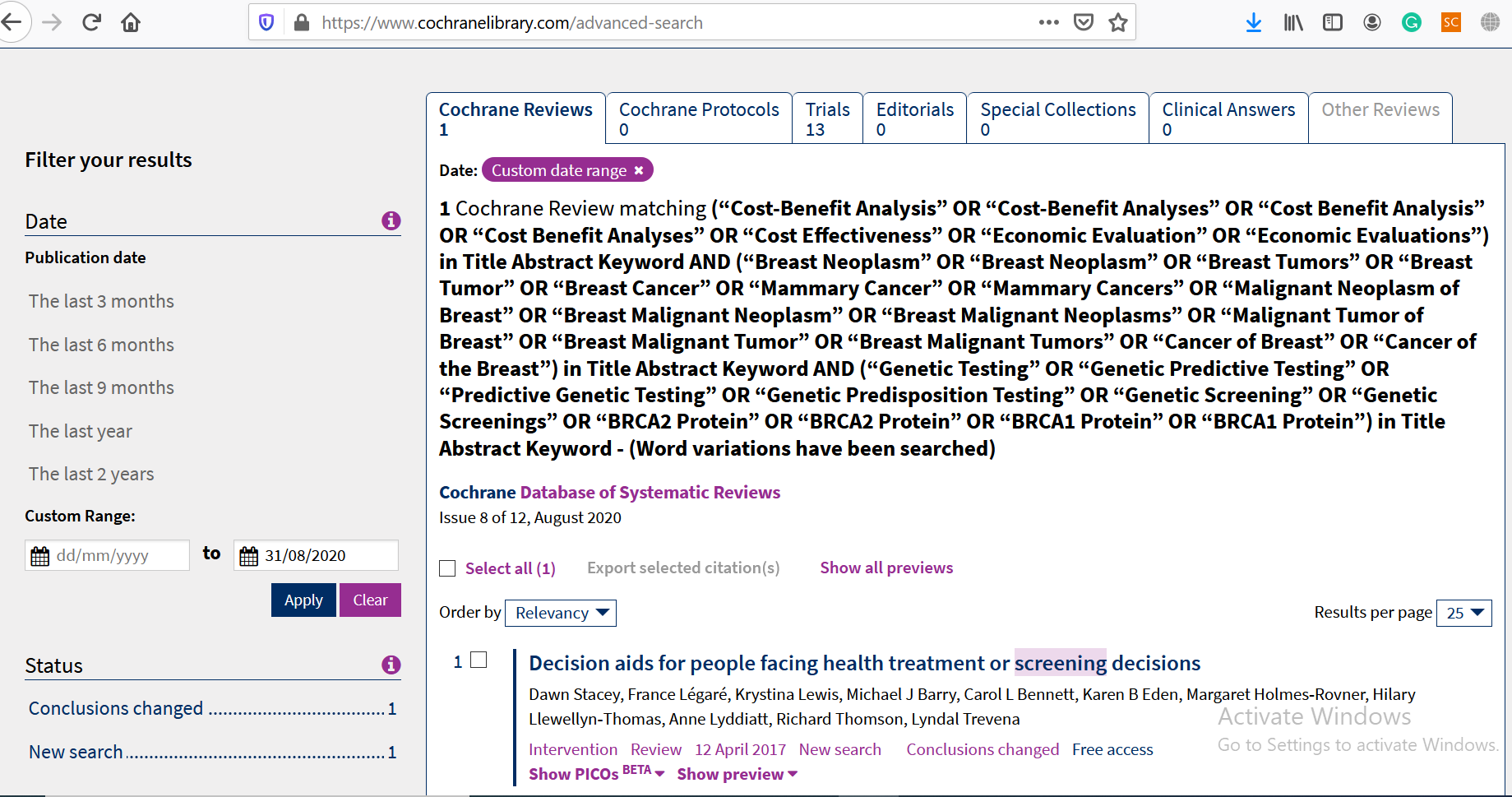


##
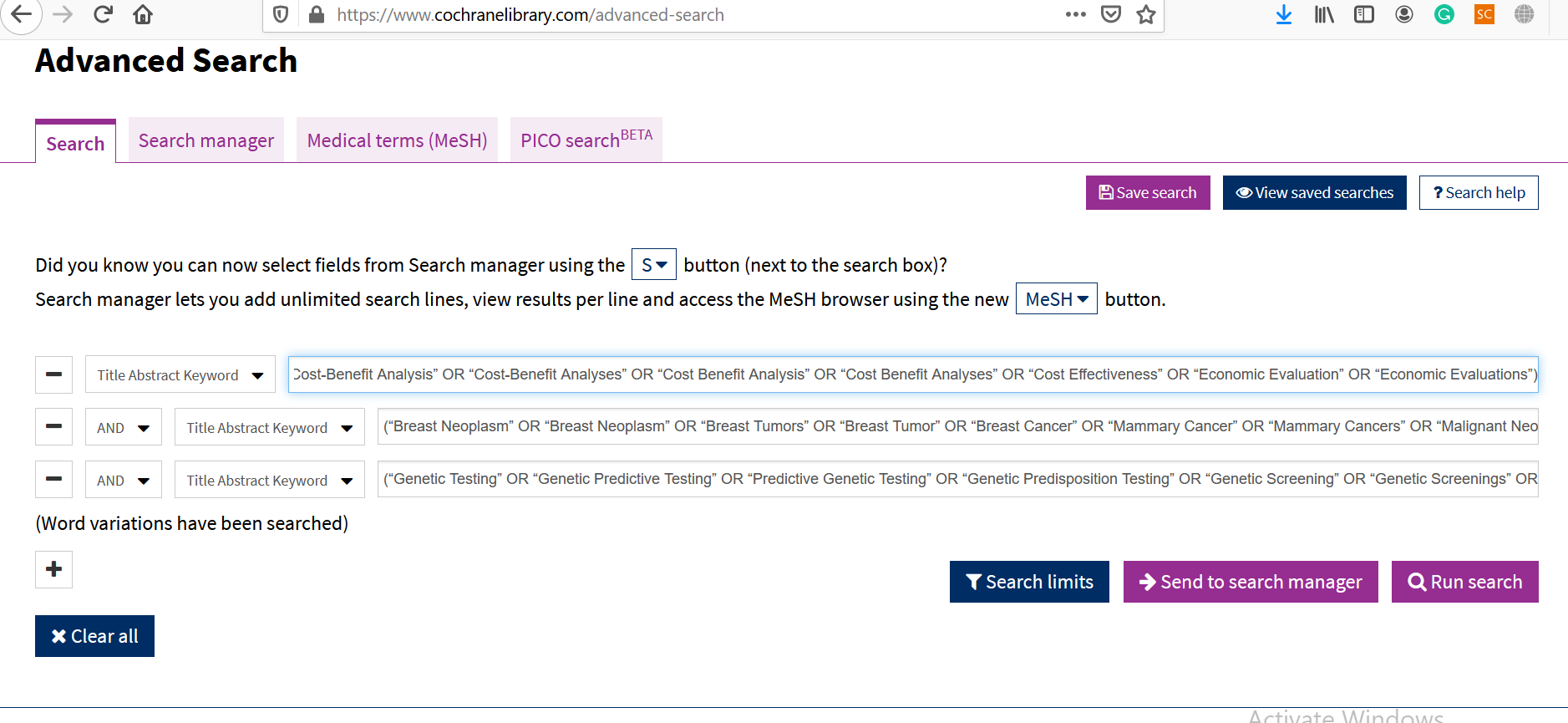

Supplement: Supplementary file 1 — Additional file 1. [file 13053_2021_191_MOESM1_ESM.docx]
